# Selecting Temperature for Protein Crystallization Screens Using the Temperature Dependence of the Second Virial Coefficient

**DOI:** 10.1371/journal.pone.0017950

**Published:** 2011-03-30

**Authors:** Jun Liu, Da-Chuan Yin, Yun-Zhu Guo, Xi-Kai Wang, Si-Xiao Xie, Qin-Qin Lu, Yong-Ming Liu

**Affiliations:** Key Laboratory for Space Bioscience and Biotechnology, School of Life Sciences, Northwestern Polytechnical University, Xi'an, Shaanxi, People's Republic of China; University of South Florida College of Medicine, United States of America

## Abstract

Protein crystals usually grow at a preferable temperature which is however not known for a new protein. This paper reports a new approach for determination of favorable crystallization temperature, which can be adopted to facilitate the crystallization screening process. By taking advantage of the correlation between the temperature dependence of the second virial coefficient (*B*_22_) and the solubility of protein, we measured the temperature dependence of *B*_22_ to predict the temperature dependence of the solubility. Using information about solubility versus temperature, a preferred crystallization temperature can be proposed. If *B*_22_ is a positive function of the temperature, a lower crystallization temperature is recommended; if *B*_22_ shows opposite behavior with respect to the temperature, a higher crystallization temperature is preferred. Otherwise, any temperature in the tested range can be used.

## Introduction

After the successful accomplishments of the Human Genome Project (HGP), more and more scientists have concentrated considerable interest on solving molecular-based diseases, which can be treated by structure-based rational drug design. Obtaining the 3-dimensional structure of the target biomacromolecules, which are often proteins, is the key to success in achieving this goal. Due to the potentially important applications of the structural information in human health, researchers have been making broad efforts to investigate the structures and functions of many proteins. It is well known that X-ray crystallography is the most widely used method to determine the 3-dimensional structure of proteins. More than 85% of the structures in the PDB (www.pdb.org) were determined by this method, which requires high quality protein crystals as diffraction targets. However, due to the complexity in crystal nucleation and growth, obtaining satisfactory protein crystals is often the rate-limiting step for structure determination. For example, over 60% of the targets for most commercial therapeutic drugs are membrane proteins [1], [2], which are usually hard to crystallize. Therefore, growing high quality protein crystals is an important task for structural biologists [3], [4].

Generally, it is accepted that if we could understand more clearly the behaviors of crystal nucleation and growth under various conditions, we would better know how to proceed with crystallization. To determine the structure of a protein using X-ray crystallography, the first step (after purification of the protein) is to find suitable conditions for crystallization (crystallization screening). Then, based on the screening results, the goal is to optimize the crystal quality for the purposes of high resolution diffraction. Since there are still no general guidelines for growing high quality protein crystals, these steps are often based on trial and error, which consumes time, money and manpower. To expedite the process and reduce the cost, rational protein crystallization has been proposed, and many efforts have been made [5]–[13]. Among these efforts, DLS (Dynamic Light Scattering) and SLS (Static Light Scattering) are often used. A famous coefficient of a protein solution, the second viral coefficient (*B*_22_), is familiar to protein crystal growers [14]–[16] and is derived from the SLS method. *B*_22_ is a static parameter that is related to the molecular weight of the protein [17]. For protein crystal growers, the *B*_22_ value is a useful parameter because it can indicate which solutions are not favorable for crystallization. A “crystallization slot” of *B*_22_, which is in the range of about −1×10^−4^ to −8×10^−4^ mol⋅mL⋅g^−2^ [18], has been reported to be helpful. A *B*_22_ value in this range does not guarantee successful crystallization, but a value outside of the slot will probably result in crystallization failure. By varying protein solution conditions (such as adding crystallization agents and additives or adjusting pH), *B*_22_ values may be adjusted to fall well within the slot, which may favor crystallization [19], [20].

It is now clear that the absolute values of *B*_22_ are affected by many factors, such as the characteristics of the particles in the protein solution, pH, intermolecular surface potential and temperature [21]. Probing the relationship between *B*_22_ and these factors may help to develop a rational strategy to determine the best crystallization parameters. For example, it has been reported that the temperature dependence of *B*_22_ shows the same tendencies as the temperature dependence of the solubility [22], [23]. This result implies that the temperature dependence of *B*_22_ could be a clue to select a suitable crystallization temperature because the driving force of crystallization is strongly related to the solubility.

The driving force of protein crystallization is the difference in chemical potential between the supersaturated and equilibrium solutions, which is mainly determined by the supersaturation (σ), i.e., the ratio of the solution concentration to the solubility (***s***). Therefore, the solubility is a crucial parameter for protein crystallization. Because the solubility usually depends on the temperature, it can be adjusted by changing the temperature. The driving force of the crystallization can thus be adjusted by selecting a suitable temperature. In routine protein crystallization screens, researchers usually choose the crystallization temperature arbitrarily because they don't have a better method. For example, a typical temperature, like 277K, is often used. If we have information about how the solubility changes in relation to temperature, which can be inferred from the results of a *B*_22_ temperature dependence measurement, we may rationally propose a suitable temperature for crystallization screens.

To demonstrate this idea, we measured the temperature dependence of *B*_22_ for lysozyme, proteinase K, concanavalin A and *α*-chymotrypsinogen A(II) in different solution conditions respectively (detailed solution conditions are listed in Table 1), and verified with reproducibility and crystallization screening studies that optimal temperatures can be selected according to the temperature dependence behavior of *B*_22_.

**Table 1:** Solution conditions for measuring B_22_.

A)		
Proteins	Buffer	
Lys.	25 mM HEPES-Na, pH 7.0	
Pro. K	25 mM HEPES-Na, pH 7.0	
Chy. A	25 mM HEPES-Na, pH 7.0	
Con. A	25 mM HEPES-Na, pH 7.0	

B)		
Proteins	Buffer	Crystallization agents
Lys.	20 mM NaAc, pH 4.6	12 mg/mL NaCl
Pro. K	5 mM HEPES-Na, pH 7.0	10 mM sodium cacodylate trihydrate; 16 mM MgAc_2_ and 5%w/v PEG8000, pH 6.5
Chy. A	20 mM citric acid, pH 3.5 and 2.5%w/v PEG3350	5%w/v PEG3350
Con. A	5 mM HEPES-Na, pH 7.0	20 mM Tris-HCl and 1.6%w/v PEG8000, pH 8.5

A): in the absence of crystallization agents; B): in the presence of crystallization agents. Lys.: lysozyme; Pro. K: proteinase K; Chy. A: α-chymotrypsinogen A (II); Con. A: concanavalin A.

## Materials and Methods

### Materials and experimental instruments

Four proteins were used in this study. Hen egg white lysozyme (HEWL, Lot No. 100940, recrystallized six times) was purchased from Seikagaku Kogyo Co. (Japan), and proteinase K (Lot No. P6556), *α*-chymotrypsinogen A(II) (Lot No. C4879) and concanavalin A (Lot No. L7647) were purchased from Sigma-Aldrich Co. (USA).

Sodium chloride (NaCl) was obtained from Tianjin Kermel Chemical Reagents Development Center (China). Sodium acetate and HEPES-Na [C_8_H_17_N_2_O_4_SNa] were obtained from Beijing Chemical Factory (China). Both acetone and toluene were analytical reagents from Henan Mol Chemical Co., Ltd. (China). Acetic acid (HPLC grade) was obtained from TEDIA Co. (USA). The crystallization screening kit was Index™ from Hampton Research Co. (USA). Sodium cacodylate trihydrate, Polyethylene glycol (PEG) 8000 and PEG 3350 were purchased from Sigma-Aldrich Co.(USA). Magnesium acetate was taken from Tianjin Chemical Reagent Co., Ltd. (China). Citric acid was obtained from Tianjin Dongli Chemical Reagent Factory (China). Tris hydrochloride (Tris-HCl) was purchased from Shanghai Fanke Biotechnology Co., Ltd (China).

Measurement of pH was carried out using a digital pH meter (Sartorius PB-10, Sartorius Scientific Instruments Co., Ltd., Beijing, China.). Water was prepared using a Nanopure Diamond Ultrapure Water System D11931 from Barnstead Co. (USA).

All prepared solutions were filtered through 0.1 µm low protein binding non-pyrogenic syringe filters (PN: 4611) from Pall China (Beijing, China).

The PCS8501-glass cuvette with round aperture, the container for *B*_22_ measurement, was obtained from Malvern Company (Beijing, China).

In the reproducibility study, 40-well plates (Keyu Co., Jiangsu, China) were used as crystallization plates, and 96-well crystallization plates (HR3-143, Hampton Research Co., USA) were used in crystallization screens.

The refractive index of protein solutions was measured by an Abbe Refractometer (Shanghai Changfang Optical Instruments Co., LTD, China). Weight measurements were carried out using a microbalance BS 224S (Sartorius AG Beijing, China). The sample concentration after light scattering measurement was detected by a UV Spectrophotometer U-3310 (Hitachi Technologies Co., Japan). *B*_22_ was measured by a Nano Zetasizer (Nano-ZS, Malvern Instruments Ltd., UK).

The crystallization trials were set up using an automated protein crystallization robot (Screenmaker 96+8, Innovadyne Technologies Inc. USA).

The resulting samples were examined by an automated crystal image reader (XtalFinder, XtalQuest Inc., China).

### Measurement of *B_22_*

#### Principle

The molecular weight and the second virial coefficient of a protein sample in a solution can be measured using the static light scattering (SLS) technique. SLS data are analyzed using the classical Zimm equation [24], [25]:
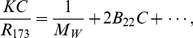
(1)where *C* is the concentration, *R*_173_ is the excess Rayleigh factor at a scattering angle of 173°, *M_w_* is the molecular weight, and *K* is an optical constant given by
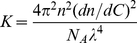
(2)where *n* is the refractive index, *N_A_* is Avogadro's number and λ is the wavelength of the detecting light.

Using the above equations, the Debye plot, which is the dependence of the solution's scattering intensity on the concentration, can be plotted. Both *B*_22_ and *M_w_* can then be derived simultaneously from the Debye plot. A detailed method can be found in the literature [26].

#### Measurements

Determination of protein concentration: an accurate protein concentration is important to calculate *B*_22_. The concentration levels of the proteins are preset to 0.5 mg/mL, 1.0 mg/mL, 3.0 mg/mL, 5.0 mg/mL, 7.0 mg/mL and 9.0 mg/mL. However, the exact concentration will normally deviate from the preset point. Therefore, to obtain reliable concentration data, we used the calculated concentration based on the actual values of solution volume and protein weight during the experiment.

Determination of *dn/dC*: from Eq. (2), the *dn/dC* value should be determined prior to the SLS measurement. We used an Abbe Refractometer to measure the refractive index at different concentration levels. Then, the *dn/dC* value was derived from the data using a linear regression treatment [27].

Cleaning of quartz sample cells: to obtain highly precise results in DLS and SLS measurements, the sample solution should be free of dust. If there is dust in the quartz sample cells or dirt on the cell wall, the measurement results will be scattered. Therefore, cleaning the quartz sample cells is very important.

We used the following cleaning procedure, which proved to be useful to enhance measurement reproducibility. First, wash the sample cell twice using distilled water and wipe with cotton swabs; then, rinse and dry the cells in a vacuum oven at 308K. Next, spray the sample cell with acetone or ethanol by using a syringe. Finally, wrap the sample cell with aluminum foil, which has itself been dunked in acetone to remove dust, to keep dust from entering the cell. Dry the cells in the ambient environment.

SLS & DLS measurements: we used SLS measurements to obtain *B*_22_ from the Debye Plot at different temperatures. Simultaneously, we performed DLS measurements using the same system. The DLS measurements were used to characterize the particle size distribution in the tested solution so as to get information on solution dispersity. To get a reliable Debye Plot, the solution should be monodisperse. If the DLS measurements show that the solution is not monodisperse, the SLS data must be discarded, and the experiments performed again.

### Crystallization screening and crystallization reproducibility tests

According to the results of the temperature dependence of *B*_22_, we may postulate about the temperature dependence trends of the solubility. In low solubility conditions, a high probability of crystallization or precipitation might be achieved. To verify this postulate, we carried out both crystallization screening and crystallization reproducibility tests. In the crystallization screening tests, four proteins were tested at the following temperatures: 277K, 289K and 301K. All proteins are dissolved in 25 mM pH 7.0 HEPES-Na. Initial protein concentrations (before mixing) were 20 mg/mL for lysozyme, 20 mg/mL for *α*-chymotrypsinogen A(II), 10 mg/mL for concanavalin A and 30 mg/mL for proteinase K [17], [28]. In the crystallization reproducibility tests, one protein (*α*-chymotrypsinogen A(II)) was tested at 277K and 293K. For comparison, crystallization reproducibility data for the other three proteins were extracted from previously published results.

## Results and Discussion

### Measurement results of *B_22_*

#### Concentration dependence of refractive index (dn/dC)

**Figure 1:**
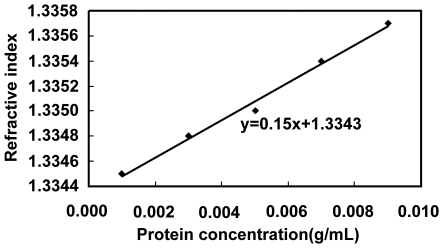
Linear regression of refractive index versus concentration of lysozyme (*dn/dC*). It can be derived that the *dn*/*dC* value is equal to 0.15 mL/g for all tested proteins in a buffer of 25 mM pH 7.0 HEPES-Na.

The refractive indices of the four proteins were measured at different concentration levels. Nearly the same measurement results were obtained for all tested proteins. Fig. 1 gives the measurement results for lysozyme. From the figure, it can be derived that the value of *dn/dC* was about 0.15 mL/g for proteins dissolved in a buffer of 25 mM pH 7.0 HEPES-Na. This value has been proven to be stable in the buffer and insensitive to the temperature change in the range between 277K and 303K. Therefore we used 0.15 mL/g as the value of *dn/dC* in the SLS measurement.

#### Concentration after SLS measurement

To make sure that the concentration data used in the SLS measurements are correct, we measured the concentration of the proteins using a UV Spectrophotometer after the SLS measurement. The results showed that there are only subtle differences between the measured results and the calculated results using the actual solution volume and weight of the proteins.

#### Obtaining a reliable Debye plot

A Debye plot is constructed by passing incident light through the protein solution and measuring scattered light intensity to see how the light interacts with the particles in the solution. Dust with diameters between 1 µm and 10 µm, which is about 100 times the size of the protein molecules, can ruin the experiment. By using a 0.1 µm filter, we successfully avoided introducing dust into the solution during solution preparation.

**Figure 2:**
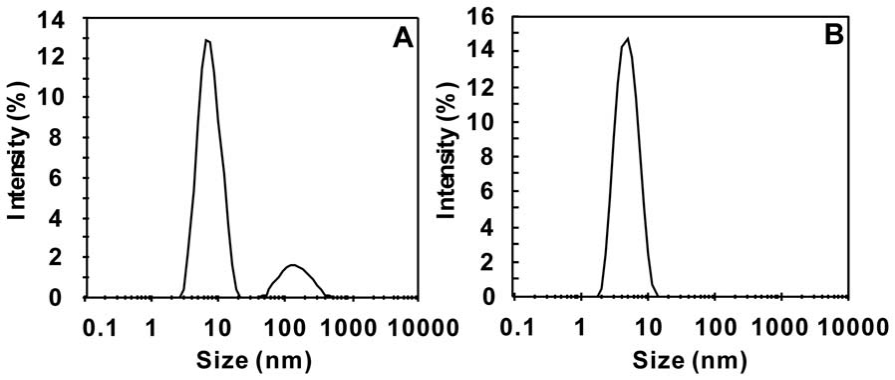
DLS measurement results for the particle size distribution of the tested solutions of *α*-chymotrypsinogen A(II). All proteins are dissolved in 25 mM pH 7.0 HEPES-Na at 295K. A): Two peaks appeared, showing that the solution was not monodisperse; B): Only one peak appeared, showing that the solution was monodisperse, and its corresponding SLS measurement result was considered reliable and the data could be included for subsequent analysis.

However, it is still very hard to completely prevent dust particles in the air from entering the solution when transferring the protein solution into the sample cell. Therefore, to make sure that the measurement of *B_22_* is not affected by the dust particles in the solution, we performed a DLS measurement at the same time to check the monodispersity of the solution. Figs. 2*(a)* and *(b)* show examples of a DLS measurement of particle size distribution in the tested solution. From the figure, we can see that the solution presented in Fig. 2A was not monodisperse; therefore, we discarded the SLS measurement data for the corresponding solution. In Fig. 2B, the solution is monodisperse and thus the results of the SLS measurement were considered reliable and safe to use.

#### The temperature dependence of B_22_

**Figure 3:**
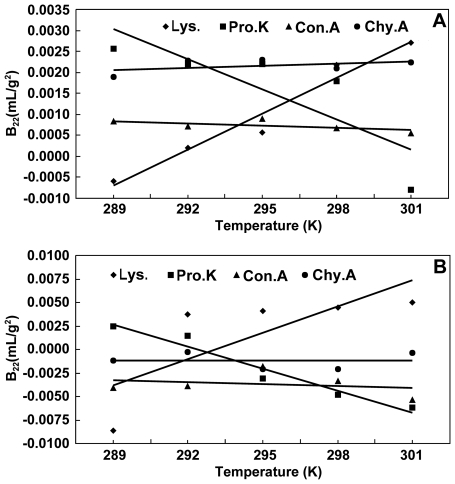
Temperature dependence of *B_22_* for the tested proteins. A): without crystallization agents; B): with crystallization agents. Refer to Table 1 for detailed solution conditions. Lys.: lysozyme; Pro.K: proteinase K; Con.A: concanavalin A; Chy.A: *α*-chymotrypsinogen A(II).

The temperature dependence of *B*_22_ in several proteins was obtained by measuring *B*_22_ at different temperatures for each protein. Fig. 3A shows the measurement results when the proteins were dissolved in the buffer only (i.e., without crystallization agents). It can be seen that lysozyme showed a “normal” temperature dependence of *B*_22_, i.e., *B*_22_ increases with increasing temperature in the tested temperature range. Proteinase K, however, showed an opposite temperature dependence of *B*_22_. The value of *B*_22_ for the other two proteins, concanavalin A and *α*-chymotrypsinogen A(II), seemed not to be very sensitive to the temperature, though *B*_22_ of the former decreased slightly with increasing temperature while that of the latter increased slightly with the temperature. Fig. 3B shows the measurement results when crystallization agents were used in the solutions. Although the absolute values of *B*_22_ presented in Fig. 3B appears smaller than their counterparts in Fig. 3A, the temperature dependence of *B*_22_ showed the same tendency against the temperature. Obviously, this result indicated that testing the temperature dependence of *B*_22_ can be carried out without using crystallization agents.

### Crystallization studies

As reported in the literature [15], [21], [23], [29]–[31], the temperature dependence of *B*_22_ can be an indication of the temperature dependence of the solubility. This rule may be used to guide the crystallization screens of proteins because the solubility is closely related to the supersaturation, which is the driving force of crystallization. A “normal” behavior of *B*_22_ against temperature indicates a “normal” behavior of solubility versus temperature, i.e., the solubility increases with the temperature. In such cases, a solution at a certain concentration will exhibit a higher supersaturation level at lower temperature. The solution will thus exhibit a high driving force for the crystallization, which is beneficial for enhancing the success rate of crystallization. To examine this speculation, we carried out crystallization studies using the four proteins whose *B*_22_ values had been measured. Both crystallization screening and crystallization reproducibility tests were used in this study.

#### Crystallization screening tests

For the screening tests, we used the two temperatures of 289K and 301K, which marked the ends of the tested temperature range in our *B*_22_ measurement. To further check the crystallization outside the above temperature range, we also tested the crystallization screens at a lower but frequently used temperature of 277 K.

**Figure 4:**
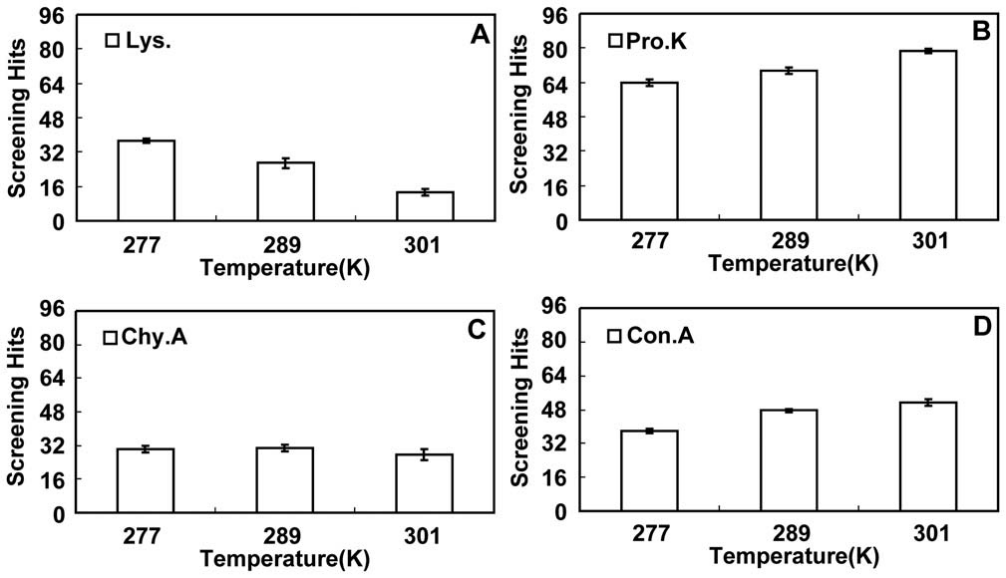
Crystallization screening hits for the tested proteins at different temperatures. The proteins used were as follows: A): lysozyme; B): proteinase K; C): α-chymotrypsinogen A(II); D): concanavalin A. The screening kit was Index™ from Hampton Research. The results showed that a protein yielded higher crystallization success rate at the temperature where the *B_22_* value of the solution was relatively lower. Error bar: standard error mean; n = 6.

Fig. 4 shows the results of the crystallization screening tests. It can be seen that, in the case of crystallization of lysozyme, the number of screening hits (defined as the number of droplets out of the 96 crystallization conditions that yielded detectable crystals under a stereomicroscope at 80×magnification) was higher at lower temperature. The difference in screening hits at different temperatures was clear: the screening hits were 24.8% greater at 277K than at 301K. In the case of crystallization of *α*-chymotrypsinogen A(II), we also observed similar trends in screening hits as seen in lysozyme crystallization, but the difference in screening hits for this protein was not very obvious: the screening hits were only 4.7% greater at 277K than at 301K. Thus, the change was relatively insensitive to the temperature just as the temperature variation of *B*_22_ for *α*-chymotrypsinogen A(II) was small compared with that for lysozyme.

As expected, in the case of crystallization of proteinase K, which exhibits opposite behavior of *B*_22_ versus temperature, the screening hits were greater at higher temperature. In the case of crystallization of concanavalin A, we observed a similar trend to proteinase K (screening hits were 15.5% greater at 301K than at 277K for proteinase K and 14.1% greater for concanavalin A), though the *B*_22_ value of the latter only slightly decreased with increasing temperature.

**Figure 5:**
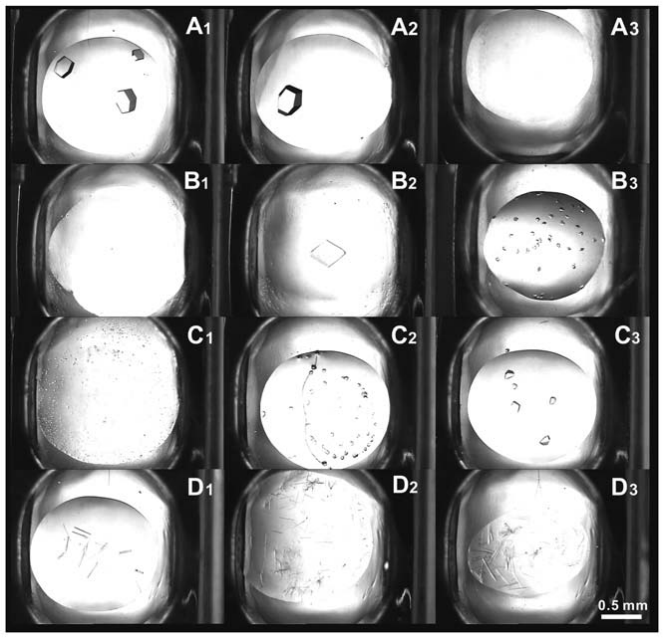
Comparison of typical crystal images of the tested proteins at screening temperatures of 277K, 289K and 301K. The screening kit was Index™ from Hampton Research. A_1_, A_2_, A_3_: lysozyme crystals obtained at C7; B_1_, B_2_, B_3_: proteinase K crystals obtained at B10; C_1_, C_2_, C_3_: concanavalin A crystals obtained at H8; D_1_, D_2_, D_3_: *α*-chymotrypsinogen A(II) crystals obtained at G6. Initial concentration of all proteins was 20 mg/mL. A_1_, B_1_, C_1_, D_1_: 277K; A_2_, B_2_, C_2_, D_2_: 289K; A_3_, B_3_, C_3_, D_3_: 301K. This figure shows that the crystal number varied with crystallization temperature as predicted by the measurement of B_22_.

Fig. 5 shows a comparison of typical crystal images for each of the tested proteins at the three screening temperatures. From the figure it can be seen that the crystal number showed the tendency against the crystallization temperature as predicted by B_22_ measurement.

#### Crystallization reproducibility tests

It is well known that protein crystallization often suffers from the problem of bad reproducibility [32], [33], i.e., identical crystallization conditions may not yield identical crystallization results. By setting up a number of crystallization drops at identical crystallization conditions, we can easily check the reproducibility of the crystallization of a protein. The major application of a reproducibility test in this study is to statistically clarify the trend of the crystallization success rate versus the temperature.

**Figure 6:**
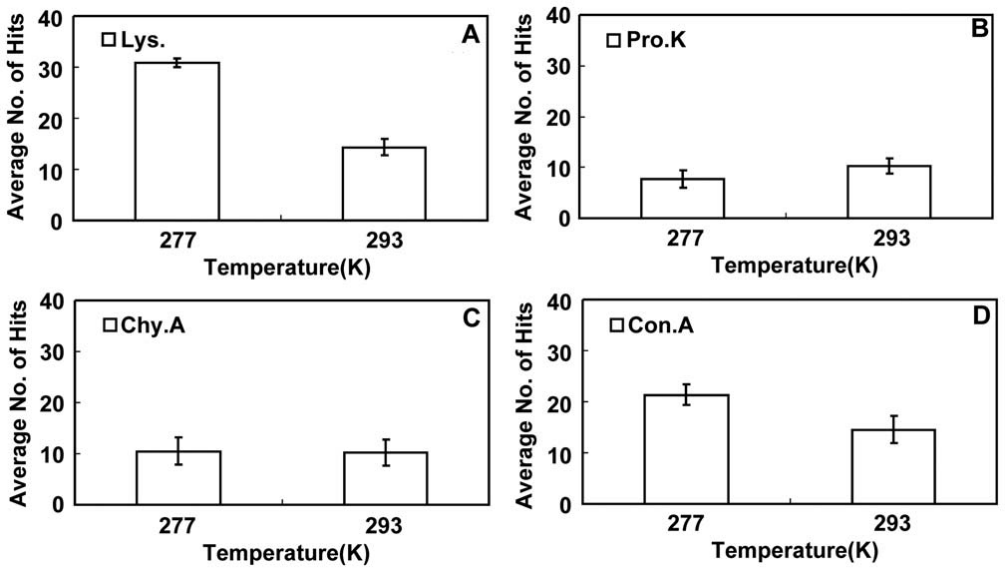
Crystallization reproducibility tests of the four proteins at temperatures of 277K and 293K. Part of the data (Fig. 6A, B and D) in this figure were extracted from published results [34] (n = 7). Crystallization methods: hanging drop. Initial crystallization conditions: A): lysozyme (Lys.) solution: 20 mg/mL in 0.1 M sodium acetate (pH = 4.6), reservoir solution: 60 mg/mL NaCl; B): proteinase K (Pro.K) solution: 20 mg/mL in 25 mM HEPES-Na (pH = 7.0); reservoir solution: 50 mM sodium cacodylate trihydrate, 80 mM Mg(Ac)_2_ and 25% w/v PEG 8000 at pH = 6.5; C): *α*-chymotrypsinogen A(II) (Chy.A) solution: 20 mg/mL *α*-chymotrypsinogen A(II) in 0.1 M citric acid (pH = 3.5) and 12.5% w/v PEG 3350; reservoir solution: 25% w/v PEG 3350; D): concanavalin A (Con.A) solution: 20 mg/mL in 25 mM HEPES-Na (pH = 7.0); reservoir solution: 0.1 M Tris-HCl and 8% w/v PEG 8000 at pH = 8.5.

In our recent publications, we have already presented some reproducibility studies at two temperatures (277K and 293K) [34], which can be used in the current study to show the trend of crystallization success rate versus the temperature. We extracted the data for reproducibility tests of three proteins (lysozyme, proteinase K, and concanavalin A) from our previous publication [34] and carried out a new crystallization reproducibility test of *α*-chymotrypsinogen A(II). Fig. 6 shows the results. From the figure, we can see that the crystallization success rate of these four proteins follows the same trends versus temperature as seen in the above crystallization screens section. The trend was especially clear in the cases of crystallization of lysozyme (Fig. 6A) and concanavalin A (Fig. 6D). In the case of *α*-chymotrypsinogen A(II) (Fig. 6C), the trend was not so clear; this result was similar to the results obtained in the screening tests (Fig. 4C). In conclusion, the crystallization reproducibility studies shown here confirm the results obtained in the screening tests.

From different sources in the literature, we also found an example which supports our hypothesis. Wilson et al. [18] reported that *B*_22_ of thaumatin I exhibits no temperature dependence, which implies an insensitive temperature dependence of its solubility. In our previous publication [33], we presented a variable temperature strategy to screen the crystallization conditions, which showed that the crystallization success rate of thaumatin is not sensitive to the temperature. This result implied that the solubility of thaumatin is insensitive to the temperature too. Apparently, the above two experimental results are in good agreement with each other and can be used as a good example to confirm our current research results.

### Potential application in protein crystallization screens

As demonstrated above, the results of the crystallization studies were clearly in agreement with the theoretical speculation. In other words, knowing the temperature dependence of *B*_22_ may be a good tool to select a suitable temperature for protein crystallization screens. The guideline could be as follows: when *B*_22_ is lower at a lower temperature (indicating a lower solubility at lower temperature), a lower screening temperature, e.g., 277K, is preferred; when *B*_22_ is higher at a lower temperature (indicating a lower solubility at higher temperature), a higher screening temperature is preferred, depending on the crystallization method and the protein. For example, only at 318K can crystal growers obtain the diffraction-quality crystals of an antifreeze protein [35]. In the rare case where *B*_22_ is insensitive to temperature, any temperature in the range where the protein is stable can be used.

There is a practical consideration about the consumption of the protein to address before this method can be applied in protein crystallization screens. In our current research, we used a normal cuvette to measure *B*_22_, which requires 1.2 mL of protein solution for each single measurement. Therefore, to obtain a complete temperature dependence of *B*_22_ with this method, approximately 26 mg of protein is necessary. This amount is too large to use in actual crystallization because proteins are usually very precious and difficult to obtain in large amounts (to ensure the homogeneity of the sample). Fortunately, if we use the Low-volume quartz batch cuvette ZEN2112 (Malvern Instruments Ltd., UK), the total amount of protein consumed can be reduced to less than 2 mg. The amount of consumed protein can be further reduced to 30-300 µg by using a light scattering technique developed to analyze droplets because droplet volume can be reduced to less than 1 µL [10]. In such cases, it would be easy to apply the method without any large sample consumption.

### Conclusions

In this paper, we presented an alternative method to help crystal growers determine a favorable temperature for protein crystallization screens by using knowledge of the temperature dependence of the second virial coefficient *B*_22_. This information is a good indicator of the temperature dependence of the solubility of the protein. By using this method, we examined the crystallization success rate of four proteins in both crystallization screening and crystallization reproducibility studies. We verified that the temperature dependence of *B*_22_ may be used as an indicator to choose a favorable crystallization temperature, which can help to increase the crystallization success rate.

The temperature for protein crystallization screens is usually chosen arbitrarily in most labs. When a crystallization screening experiment yields no hits, it is hard to know if the cause was an unsuitable temperature or unsuitable solution conditions. If the chosen temperature is shown to be unsuitable after a couple of trials, it must be changed to other temperatures using only trial and error tests, which will waste time, money, and protein before a suitable temperature is found. Therefore, determining a more favorable temperature in advance will be very useful for carrying out the crystallization screens more easily and efficiently. The method proposed in this paper was proven effective, and we recommend it as an alternative method to rationally find a suitable temperature for protein crystallization screens.
